# Germline *PALB2* Mutations in Cancers and Its Distinction From Somatic *PALB2* Mutations in Breast Cancers

**DOI:** 10.3389/fgene.2020.00829

**Published:** 2020-08-27

**Authors:** Zhe-Yu Hu, Liping Liu, Ning Xie, Jun Lu, Zhentian Liu, Yu Tang, Yikai Wang, Jianbo Yang, Quchang Ouyang

**Affiliations:** ^1^Affiliated Cancer Hospital of Xiangya Medical School, Central South University/Hunan Cancer Hospital, Changsha, China; ^2^Department of Breast Cancer Medical Oncology, Hunan Cancer Hospital, Changsha, China; ^3^Geneplus-Beijing Institute, Beijing, China; ^4^Department of Biostatistics and Bioinformatics, Emory University Rollins School of Public Health, Atlanta, GA, United States; ^5^Fujian Medical University Union Hospital, Fuzhou, China; ^6^Department of Otolaryngology, Medical School, University of Minnesota, Minneapolis, MN, United States

**Keywords:** germline *PALB2* mutation, hereditary breast cancer, loss of heterozygosity, somatic mutations, mutational signature

## Abstract

*PALB2* is an important BRCAx candidate for familial breast cancers (FBC). *PALB2* pathogenic variants (PVs) may not to conform to “two hit” paradigm. However, a recent study demonstrates that in the majority *PALB2* germline mutant breast cancers, the loss of heterozygosity (LOH) and somatic point mutations are the “second hit.” This study aimed to investigate the second hits in germline *PALB2* mutations in breast cancers. We screened out 28 germline *PALB2-*mutation carriers among 480 familial cancer patients (including 143 FBC patients) in Geneplus database pool. Of the 143 patients with FBC, 10 had mono-allelic *PALB2* germline mutations. All these germline *PALB2* mutations were high-risk stop-gain, frameshift, or splicing mutations that concentrated in EX5–EX9 and might led to truncated proteins, severe functional defects and malignant phenotype. The hotspots were c.1057A[3 > 2] and c.3114-1G > A. Other mutations included c.389delA, c.2068C > T, c.2167_2168delAT, c.2629delT and c.2968G > T. Only one FBC patient has *PALB2* somatic mutation and two patients had LOH of *PALB2*. All germline *PALB2* mutations were high-risk mutations, whereas the somatic *PALB2* mutations were moderate-risk missense mutations. We also distinguished PALB2 “novel mutations” from “reported mutations.” In conclusion, germline *PALB2* mutation should be put into the context of future screening.

## Introduction

Nearly the one-eighth of females develop breast cancer over the course of their lifetime (Owens et al., 2019). Approximately 5–27% of breast cancers are hereditary (Lichtenstein et al., 2000). *BRCA1* was the first gene identified as a susceptibility gene for hereditary breast cancer (HBC) (Torchard et al., 1994). Soon after, *BRCA2* was also identified as a susceptibility gene for HBC (Collins et al., 1995). Not all HBCs involve *BRCA1/2* mutations. 70–80% of HBCs involve non-*BRCA1/2* (*BRCAx*) mutations (Hedenfalk et al., 2003; Keeney et al., 2017). Instead, *ATM*, *CHD8*, *CDH1*, *RAD50*, *CHEK2*, and *PALB2* are found to harbor germline mutations conferring high to moderate risk for *BRCAx* HBC (Aloraifi et al., 2015; Li et al., 2017; Tavera-Tapia et al., 2017). Most of those genes are related to DNA damage repair and their mutations in embryo are thought to increase the risk of HBC by 20–80%. *BRCAx* HBCs may develop the secondary somatic *BRCA1/2* aberrations (including point mutations and hypermethylation) (Alvarez et al., 2005). In a study of 656 families, no convincing evidence has been found to verify the risk effect of the epigenetic modifier and known germline breast cancer driver gene mutations (Li et al., 2017). But in a most recent study of 524 families with germline PALB2 variants (PVs), PALB2 was confirmed as a major breast cancer susceptibility gene, and its germline PVs also associates with ovarian, pancreatic and male breast cancers (Yang et al., 2020).

In 2006, *PALB2* has been identified as the partner and localizer of *BRCA2* (Xia et al., 2006) and a susceptibility gene for HBC (Rahman et al., 2007). Overall, 35% of females carrying *PALB2* mutations are expected to develop breast cancer before 70 years of age, and 58% of carriers with a family history of breast cancer are expected to develop the disease (Antoniou et al., 2014). By binding to the BRCA1 and BRCA2 proteins, PALB2 protein facilitates homologous recombination repair (HRR) for DNA double strand breaks (DSBs) (Ripperger et al., 2009; Wiltshire et al., 2020).

The “second hit” theory of tumor-suppressor genes (TSGs) suggests that the loss of function (LOF) mutations on both alleles of a given TSG are necessary for tumorigenesis (Knudson, 1971; Smith et al., 2016). For example, some HBC patients have mutations in both *PALB2* alleles (*PALB2-NULL*), of which one is inherited and the other is a somatic point mutation and epigenetic modification (Potapova et al., 2008; Bouwman et al., 2011; Scott et al., 2016; Lee et al., 2018). However, in some cases, *PALB2* seems not to conform to that theory. For example, some HBC patients have one heterozygous germline *PALB2* mutation (*PALB2*-HET), with one normal wild-type *PALB2* allele. Interestingly, there was no significant difference in the HRD scores between PALB2 heterozygotes and null tumors (Lee et al., 2018). These *PALB2*-HET HBC patients have more defects in homologous recombination repair (HRR) than patients with sporadic breast cancer. In addition, HRD mutational signatures are predominant in some PALB2 heterozygous carriers. The fact that most the *PALB2*-HET BC tumors also exhibit known cancer drivers suggests either tumor evolution or this demonstrates the well known phenomenon of differential positive selection (Martincorena et al., 2017).

In the haploinsufficient paradigm, malignancy can be induced by the mutation on one allele of a dose-dependent TSG (Gilad et al., 2010). In a mouse model, homozygous *PALB2* knockout is lethal; malignancies are only developed in heterozygous *PALB2* knockout mutants (Bouwman et al., 2011). In an Australian study (Lee et al., 2018), *PALB2*-HET patients have much more high-risk germline *PALB2* frameshift variants than *PALB2-NULL* patients (80 vs. 30%), suggesting that serious *PALB2* defects on one allele might be enough to induce malignant phenotype. In a Chinese study, high-risk loss-of-function (LOF) mutations (frameshift and splicing mutations) were detected rarely in patients with sporadic breast cancer (0.56%), but more frequently in HBC patients (1.31%) (Zhang et al., 2017). Thus, we hypothesize that heterozygous germline *PALB2* LOF mutations cause PALB2 functional haploinsufficiency, leading to HRR impairment and HBC.

## Materials and Methods

### Patient Cohort

This study was approved by the Ethics Committee at Beijing Geneplus Institute and Hunan Cancer Hospital. All patients provided informed consent for genetic analysis of their genomic DNA (gDNA) and circulating tumor DNA (ctDNA). 480 familial cancer patients (including 143 familial breast cancer patients) were assessed by specialist Familial Cancer Clinic and determined to be sufficiently strong to be eligible for clinical genetic testing by local criteria. In addition, 196 sporadic advanced breast cancer (ABC) patients without family history were also assessed. The somatic and germline pathogenic variants using a 1021-gene panel (Hu et al., 2018).

The somatic *PALB2* mutations were also investigated in 986 invasive breast cancer samples from TCGA-BRCA project and in 3,090 breast cancer samples from cBioPortal database.

### Genomic and Tumor DNA Extraction

To detect the germline PALB2 variants, gDNA was extracted from peripheral blood cells using a QIAamp DNA Blood Mini Kit (Qiagen, Hilden, Germany). Peripheral blood samples were collected in Streck tubes (Streck, Omaha, NE, United States) and centrifuged within 72 h to separate the plasma from peripheral blood cells. For somatic variants detection, ctDNA was extracted from the plasma using a QIAamp Circulating Nucleic Acid Kit (Qiagen, Hilden, Germany). Both DNA extractions were performed according to the manufacturer’s instructions, as described previously (Hu et al., 2018).

### Target Capture, Next-Generation Sequencing, and Data Analysis

Sequencing libraries of gDNA and ctDNA were prepared using the DNA Library Preparation Kit for Illumina (New England Biolabs, Ipswich, MA, United States). Custom biotinylated oligonucleotide probes (IDT, Coralville, IA, United States) covering the exons of 1,021 genes that are highly mutated in 12 common solid tumors were used for hybrid capture, as described previously (Yang et al., 2017). The Illumina HiSeq 3000 Sequencing System (Illumina, San Diego, CA, United States) was used for DNA sequencing with a 2 × 101-bp paired-end strategy, as described previously (Hu et al., 2018).

Terminal adaptor sequences were removed from the raw sequencing data. Subsequently, reads with more than 50% low-quality bases, or more than 50% undefined bases, were discarded. The remaining reads were mapped to the reference human genome (hg19) using the Burrows-Wheel Aligner (BWA)^1^ with default parameters. Picard’s Mark Duplicates tool^2^ was used to identify duplicate reads. Local realignment and quality recalibration were performed using The Gene Analysis Toolkit^3^ (GATK, version 3.4-46-gbc02625). Single-nucleotide variants and small insertions and deletions were called using the MuTect2 algorithm^4^ (version 1.1.4), and further filtration and validation were performed according to established criteria (Yang et al., 2017). The Contra algorithm^5^ (v2.0.8) was used to identify somatic copy-number alterations defined using the ratio between the adjusted depths of ctDNA and control gDNA. After automatic calling, candidate variants were manually validated using an online visualization tool^6^ (Integrative Genomics Viewer, IGV).

### Loss-of-Heterozygosity (LOH) and Mutational Signature

*PALB2* LOH was also assessed in patients cohorts. Heterozygous germline single-nucleotide polymorphisms (SNP) across the *PALB2* locus were identified similarly to a recent study (Lee et al., 2018). Alternate *PALB2* allele frequencies were determined by comparing the gDNA and ctDNA sequencing results and used to infer the *PALB2* LOH status.

Different mutational processes generate unique combinations of mutation types, termed “mutational signatures.” The mutational signatures were compared between 10 *PALB2*-associated breast tumors and 30 sporadic breast tumors (without any germline mutations or familial history of breast cancer). The somatic single-nucleotide variations were divided into six groups (T > A, T > C, T > G, C > A, C > G, C > T) and 96 subgroups according to the trinucleotide context. The mutational signatures were quantified using the deconstructSigs package (Rosenthal et al., 2016) with deconvolution methods based on the 30 mutational signatures created by COSMIC (Alexandrov et al., 2013).

### Statistic Analyses

All statistical analyses were conducted by using SAS 9.4. All tests of hypotheses were two-tailed and conducted at a significance level of 0.05 and at a marginal significant level of 0.15.

## Results

### Heterozygous Germline *PALB2* Mutations and Somatic *PALB2* Mutations in FBC Malignancies

As shown in Table 1 and Table 2, among the 143 patients with familial breast cancers (FBCs), heterozygous germline *PALB2* mutations were detected in 10 patients. c.3114-1G > A [IVS10 splicing variant] was the most frequent mutation (Table 2). c.3114-1G > A [IVS10 splicing variant] and c.1057A [3 > 2][p.K353Nfs^∗^3] mutations were common in familial breast cancer. All of the mono-allelic *PALB2* germline mutations detected were high-risk LOF mutations. In these tables, we distinguished *PALB2* “novel mutations” from the “reported mutations.”

**TABLE 1:** Germline and somatic mutations in 10 patients with FBCs and heterozygous germline *PALB2* mutations.

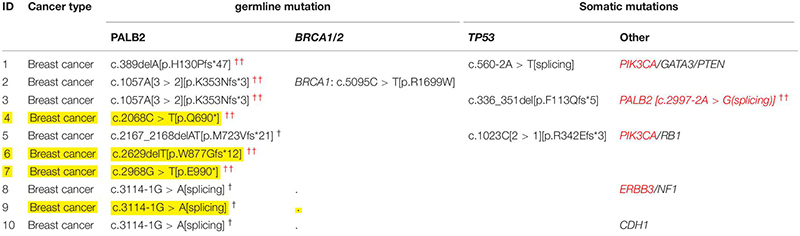

*Key somatic mutations were highlighted in red color; patients with mono-allelic *PALB2* germline mutation and without any other accompanied mutations were highlighted in yellow color. ^†^ indicated reported mutations while 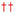 indicated the novel mutations.*

**TABLE 2:** Germline *PALB2* mutations detected in gDNA of 10 patients with FBCs.

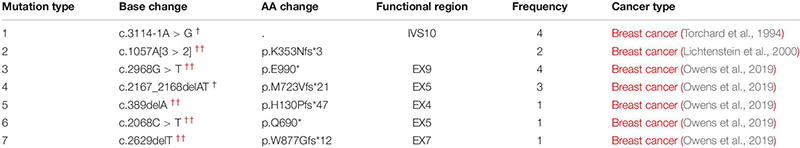

*“.” notation in the AA column indicates the amino acid mutation loci. The number in parentheses represents the number of times that the specific mutation type was detected in 143 FBC patients. ^†^ indicated reported mutations while 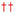 indicated the novel mutations.*

While all 18 *PALB2*-associated patients with familial cancers other than FBCs had additional somatic mutations, four of the 10 *PALB2*-associated patients with familial breast cancer had no additional somatic or germline mutations (Tables 1, 3). Compared with the other cancer types, familial breast cancer was less likely to have an additional somatic or germline mutations accompanying a germline *PALB2* mutation (6/10 vs. 18/18, Fisher’s exact test, *p* = 0.02; Table 3).

**TABLE 3:** Somatic and other germline mutations accompanied with *PALB2* germline pathogenic variants.

Accompanied mutations		Breast cancers (*n* = 10)	Lung cancers (*n* = 8)	Others (*n* = 10)	*p*-value*
Accompanied mutations (germline and somatic), n (numerator/denominator)		6 (6/10)	8 (8/8)	10 (10/10)	0.0239
Accompanied somatic mutations		5 (5/10)	8 (8/8)	10 (10/10)	0.0057
Specific Somatic mutations	*TP53*	3 (3/10)	5 (5/8)	5 (5/10)	0.42
	*PIK3CA*	2 (2/10)	0 (0/8)	2 (2/10)	0.51
	*EGFR*	0 (0/10)	4 (4/8)	0 (0/10)	0.0034
	*KRAS*	0 (0/10)	0 (0/8)	4 (4/10)	0.0239
	*APC*	0 (0/10)	0 (0/8)	4 (4/10)	0.0239
	*PALB2*	1 (1/10)	0 (0/8)	1 (1/10)	1.00

*p*-value* indicated that Fisher’s exact test was performed to calculate the *p*-value for accompanied mutations in *PALB2*-germline mutant lung cancer, breast cancer and other types of cancer patients.

Somatic *TP53* mutations were the most common type of mutation accompanying germline *PALB2* mutations. Somatic *EGFR* mutations were frequent in lung cancers, whereas somatic *KRAS* and *APC* mutations were frequent in colorectal cancers (Supplementary Table S1 and Table 3). In *PALB2*-associated breast cancers, the accompanying mutations included germline *BRCA1* mutation and somatic *TP53*, *PIK3CA*, *PALB2*, *ERBB3*, and *RB1* mutations (Tables 1, 3).

Among 143 FBCs, ten patients with familial breast cancer had heterozygous germline *PALB2* mutations, all of which were high-risk LOF mutations (Table 1). One of the 10 *PALB2-associated* patients had a somatic *PALB2* mutation; four others had somatic mutations in other genes, including *TP53*, *PIK3CA*, and *ERBB3*; one had two germline mutations (*BRCA1* and *PALB2*); and in the remaining four patients, heterozygous germline *PALB2* mutation was the only genetic event. Among 18 *PALB2* tumors other than FBCs, only one had somatic *PALB2* variant.

### Loss of Heterozygosity (LOH) and Mutation Signatures in *PALB2* Tumors

**FIGURE 1:**
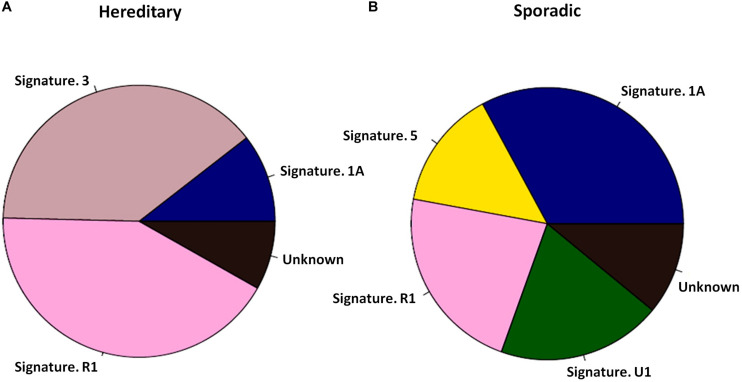
Mutational signatures of 10 *PALB2* BC tumors and 30 sporadic BC tumors. **(A)** Pie diagram exhibiting the weighted contribution of each reference signature according to COSMIC exhibiting signs of mutational signature R1 and mutational signature 3 as the largest contributors in *PALB2* BC tumors. **(B)** Pie diagram exhibiting the weighted contribution of each reference signature according to COSMIC exhibiting signs of mutational signature 1A, R1, and 5 as the largest contributors in sporadic BC tumors.

*PALB2* LOH was evaluated in 28 *PALB2*-associated tumors. Only four out of 28 tumors had true LOH at the *PALB2* allele, including 2/10 breast tumors (Supplementary Tables S2, S3). By screening ctDNA from 10 PALB2-associated HBCs and 30 sporadic ABCs, we found that the PALB2-associated tumors had a different mutational signature from the sporadic tumors (Figure 1). Mutational signature 3, which is related to defective HRR (Nik-Zainal et al., 2016), was present in 4 of PALB2-associated breast tumors, including all three PALB2-NULL BC cases, but absent among sporadic breast tumors (Supplementary Figure S1–S2). Besides the mutational signature 3, the mutational signature R1 was also common in *PALB2*-associated breast tumors.

### Somatic and Germline *PALB2* Mutations in Sporadic Advanced Breast Cancers

Six of 196 (6/196) sporadic ABC patients had heterozygous germline *PALB2* mutations, and other three patients (3/196) had somatic *PALB2* mutations (Supplementary Figure S3). The rate of heterozygous germline *PALB2* mutation was much higher than the rate of somatic *PALB2* mutation (6/196 vs. 3/196). All six germline *PALB2* mutations were high-risk LOF mutations (Supplementary Figure S3A), including two c.3114-1G > A splicing mutations, two frameshift mutations (p.W877Gfs^∗^12 and p.K353Nfs^∗^3), and two stop-gain mutations (p.E990^∗^). None of the six patients with germline *PALB2* mutations had any somatic *PALB2* mutations. In four of those patients, the heterozygous germline *PALB2* mutation was the only genetic mutation identified. In the resting two patients, one was accompanied by a germline *BRCA1* mutation; the other one had somatic mutations in other genes, such as *NF1* and *ERBB2*.

The three somatic *PALB2* mutations were moderate-risk missense mutations (p.L763F, p.E53K, and p.P5S, Supplementary Figure S3B). As shown in Supplementary Table S4, ABC patients with somatic *PALB2* mutations had high tumor mutation burden (TMB), while germline mutant patients exhibited low (even U) TMB. In follow-ups, we found that all three *PALB2* somatic mutant patients progressed within 6 months after *PALB2* somatic mutations were detected. However, among *PALB2* germline mutant patients, only one progressed within 6 months, and the rest did not progress within 6 months (Supplementary Table S4).

### Somatic *PALB2* Mutations in Public Databases

The frequency of somatic *PALB2* mutations among patients with invasive breast carcinoma in the TCGA database (TCGA-BRCA) was 1.12% (11/986; Supplementary Figure S4A). Across all cancer types, there were 209 patients in the TCGA database with a total of 158 unique somatic *PALB2* mutations, most of which (134/158) were missense mutations (Supplementary Figure S4B). High-risk frameshift mutations p.N280Tfs^∗^8 and p.M296^∗^ were the most frequent *PALB2* mutations in the TCGA database (appearing in five and six patients, respectively; Supplementary Figures S4B,C). Of the high-risk *PALB2* mutations, only the p.Q1146^∗^ stop-gain mutation appeared in TCGA-BRCA patients. Among 3,090 patients with breast cancer in the cBioPortal database, only 25 (25/3,090) had somatic *PALB2* mutations; 20 were missense mutations and five were high-risk mutations, including four stop-gain mutations (E12^∗^, E667^∗^, Q1146^∗^, and Q822^∗^) and one frameshift mutation (I1035Mfs^∗^6).

### A Pedigree Study From a Paternal Carrier

Splicing mutation c.3114-1G > A in patient ID182 was inherited from her father (male health carrier, Supplementary Figure S5), indicating that sporadic breast cancer with *PALB2* mono-allelic mutation should be recognized as hereditary breast cancer. None of patient ID18’s family members had history of malignancies. But, by testing gDNA and ctDNA mutations in her family members, her father and sister were both germline *PALB2* c.3114-1G > A heterozygote mutant healthy carrier Supplementary Figure S5. These results showed a parental heredity of germline *PALB2* heterozygote mutation in a non-familial breast cancer patient. The risk of *PALB2* c.3114-1G > A mutation carriers to have cancers was listed in Supplementary Table S5. The life-time risk of breast cancer for *PALB2* normal population is 12.4% for females. But among *PALB2* c.3114-1G > A mutation carriers, this risk increased to 33–58% for females. Germline *PALB2* c.3114-1G > A heterozygote mutation was a pathological gene in this family. Thus, even this family has no history of evidenced malignancies, patient ID18 was definitely a hereditary breast cancer patient.

## Discussion

In this study, we summarized the main findings from patient samples and database (Supplementary Figure S4). All of the detected heterozygous germline *PALB2* LOF mutations were high-risk LOF mutations, whereas most of the *PALB2* somatic mutations were moderate-risk missense mutations. The rate of heterozygous germline *PALB2* LOF mutation was much higher than the rate of somatic *PALB2* mutation. Most of the heterozygous germline *PALB2* LOF mutations were not accompanied by *PALB2* somatic mutations or LOH.

A recent study found the risk of female breast cancer in families with PALB2 pathogenic variants to be 7.18 fold higher than controls (Yang et al., 2020). PALB2 pathogenic variants significantly increased the risk of breast cancers, ovarian cancer, pancreatic cancer and male breast cancers. Other associations were excluded (e.g., Colon, prostate) or not yet evaluated (lung). In this study, the germline PALB2 aberrations in familial lung, colon or prostate cancer might be “incidental findings.” In Supplementary Table S6, all the listed PALB2 variations were with uncertain significance. Thus in these cases, it is possible that the PALB2 variant is not “causative” of the cancer. This may also reflect in the differences in the somatic landscape among cancers.

Heterozygous germline *PALB2* LOF mutations were previously shown to be associated with familial breast cancer with a prevalence of about 1% (Kurian et al., 2019). In the SEER database, *PALB2* mutations are among the prevalent pathogenic variants in breast cancer (Kurian et al., 2019). In our study, germline *PALB2* heterozygous mutations were detected in non-familial breast cancer patients (Supplementary Figure S5). Heterozygous germline *PALB2* LOF mutation c.3114-1G > A [splicing] was reported previously in sporadic breast cancer in Chinese patients (Zhang et al., 2017). This mutation was common not only in breast cancer but also in stomach carcinoma (Supplementary Table S1), suggesting a context of future screening in families with other cancers.

Hereditary cancer is caused by genetic mutations that pass from parents to children. Sometimes, hereditary cancer might be mis-identified as sporadic cancer because of a failure to recognize a mono-allelic mutation as a hereditary driver of cancer. Actually, sporadic cancers have been reported to be influenced by germline mutations or downstream effectors of susceptible germline mutations or in pathways that involve known susceptibility genes (Jazaeri et al., 2002; Potapova et al., 2008; Lolas Hamameh et al., 2017). Mono-allelic germline *PALB2* high-risk LOF mutation led to defects in homologous recombination repair (HRR) and caused breast cancer phenotype in our detected *PALB2*-associated patients, supporting the haploinsufficiency hypothesis for *PALB2*. Based this hypothesis, we recommended sporadic breast cancers with mono-allelic susceptibility gene mutations to be diagnosed as hereditary breast cancer (HBC), and also recommended their family members to take genetic screen and counseling.

Whole-exome or big-panel gene sequencing of gDNA and ctDNA can effectively detect all mutations in known cancer-susceptibility genes in patients with pathologically confirmed tumors. ctDNA testing also allows the evaluation of LOH and mutational signatures. Different combinations of somatic mutations relate to different mutational processes, termed “mutational signature.” The targeted sequencing of mutational signatures could be use to identify genetic risk factors for cancer. For example, mutational signature 3 is associated with germline *BRCA1/2* mutations and HRR deficiency (Alexandrov et al., 2015; Nik-Zainal et al., 2016). Mutational signatures 2 and 13 are found to be prominent in breast cancer (Alexandrov et al., 2013); they are related to the local hypermutation cancers, suggesting potentially implicating AID/APOBEC enzymes in cancer process. In our study, mutational signature 3 was more common in *PALB2*-associated tumors than in other tumors (Figure 1), suggesting a defect in DNA HRR machinery (Davies et al., 2017; Polak et al., 2017) induced by LOF mutations of *PALB2* (Nik-Zainal et al., 2016).

All of the heterozygous germline *PALB2* mutations that we detected in advanced breast cancers and familial breast cancers in the Geneplus cohort were high-risk LOF mutations, whereas all of the *PALB2* somatic mutations were moderate-risk missense mutations. The frequency of *PALB2*-associated mutations was much higher than that of somatic *PALB2* mutations. Most *PALB2*-HET breast cancers were not accompanied by *PALB2* somatic mutations, LOH, or hypermethylation (Lee et al., 2018), which supported the haploinsufficiency hypothesis. However, the lack of hepermethylation data was the limitation of this study.

In 2018, Lee et al. (2018) published a research to demonstrate the molecular basis of *PALB2*-associated breast cancer. In this article, authors found among 15 *PALB2*-germline mutant breast cancer patients, 10 were *PALB2*-NULL patients with both somatic and germline *PALB2* mutations and 5 were *PALB2*-HET patients with only germline *PALB2* mutations. Based on their findings, authors suggested that most PALB2-associated breast cancers comformed to “second hit” theory. However, when compared to our findings, we found that all the *PALB2*-germline mutations in our *PALB2*-associated breast cancers were high risk frameshift, stopgain or splicing mutations, while most of the *PALB2*-germline mutations in *PALB2*-NULL patients (7/10) were moderate missense mutation (Lee et al., 2018). Also, in 5 were *PALB2*-HET patients in Lee’s paper, most (4/5) *PALB2*-germline mutations were high-risk frameshift mutations, which consistent with our finding. So, we suggested that *PALB2*-associated patients with high-risk *PALB2*-germline mutations might not conform to the “second hit” theory.

In conclusion, while most of the *PALB2* somatic mutations were moderate-risk missense mutations, the heterozygous germline *PALB2* LOF mutations were high-risk LOF mutations. The heterozygous germline *PALB2* LOF mutation was also much more common than the somatic *PALB2* mutation in breast cancers. Most of the heterozygous germline *PALB2* LOF mutations were not accompanied by *PALB2* somatic mutations, LOH, or hypermethylation. Germline *PALB2* mutation should be put into the context of future screening, diagnostics and even for poly (ADP-ribose) polymerase (PARP) inhibitors treatment.

## Data Availability Statement

The CNGBdb website is https://db.cngb.org/data and the request number is CNP0001128.

## Ethics Statement

The studies involving human participants were reviewed and approved by the Ethics Committee at the Hunan Cancer Hospital and Beijing Geneplus Institute. All patients provided informed consent for genetic analysis of their genomic DNA (gDNA) and circulating tumor DNA (ctDNA). The patients/participants provided their written informed consent to participate in this study.

## Author Contributions

QO and Z-YH designed this study and interpreted the outcomes regarding surgery and survival. All authors helped perform the data analysis and prepare the manuscript and read and approved the final manuscript. Z-YH wrote the manuscript and performed the data analysis.

## Conflict of Interest

The authors declare that the research was conducted in the absence of any commercial or financial relationships that could be construed as a potential conflict of interest.

## Footnotes

1 http://bio-bwa.sourceforge.net/ (version 0.7.12-r1039)

2 https://software.broadinstitute.org/gatk/documentation/tooldocs/4.0.3.0/picard_sam_markduplicates_MarkDuplicates.php (version 1.98)

3 https://www.broadinstitute.org/gatk/ (version 3.4-46-gbc02625)

4 https://software.broadinstitute.org/gatk/documentation/tooldocs/3.8-0/org_broadinstitute_gatk_tools_walkers_cancer_m2_MuTect2.php (version 1.1.4)

5 http://contra-cnv.sourceforge.net (v2.0.8)

6 http://www.igv.org/

## Supplementary Material

FIGURE S1 Frequency plots of mutational signature of hereditary PALB2 tumors. The proportions of signature 1A (top), signature 3 (medium), and signature R1 (bottom) were 10.5%, 39.1%, and 42.2%, respectively.

FIGURE S2 Frequency plots of mutational signature of sporadic breast tumors. The proportions of signature 1A (top), signature 5 (medium), and signature R1 (bottom) were 32.9%, 14.2%, and 22.4%, respectively.

FIGURE S3 Germline (in the red dash rectangles) and somatic mutation profiles of advanced breast cancers with germline or somatic *PALB2* mutation. *BRCA1* (blue dash line) and *PALB2* (red dash line) mutations were highlighted. Dark blue represents the most commonly mutated genes, and light blue represents the least commonly mutated genes. If genes were mutated at the same frequency, they are listed in alphabetic order. **(A)** Among 196 advanced breast cancers, two triple-negative breast cancer (TNBC) tumors (ID35, ID78), two Luminal B (ID118, ID196), one Luminal A (ID182) and one Her2-positive tumors (ID96) had mono-allelic germline *PALB2* mutation. **(B)** One TNBC (ID163), one Luminal A (ID94) and one Luminal B (ID39) advanced breast cancers had somatic *PALB2* mutations.

FIGURE S4 Somatic PALB2 mutations detected in the TCGA database. **(A)** Percentages of patients affected by a total of 216 *PALB2* mutations across 23 projects in the TCGA database. A total of 209 patients were affected by *PALB2* mutations. Somatic *PALB2* mutations were detected in 8.09% of patients with uterine corpus endometrial carcinoma and 1.12% of patients with invasive breast carcinoma (TCGS-BRCA). **(B)** Numbers of patients affected by each of 158 somatic *PALB2* variations in the TCGA database. A total of 209 patients were affected by *PALB2* mutations. There were 134 missense mutations (red dots). The frameshift mutations (blue dots) p.N280Tfs^∗^8 and p.M296^∗^ were the most frequent mutations, affecting six and five patients, respectively). **(C)** The risk level associated with each mutation in B. The Frameshift and stop-gain mutations were high-risk *PALB2* variations (red dots). Missense mutations were moderate-risk variations (blue dots).

FIGURE S5 Pedigree for patient ID182. This patient had a heterozygous germline *PALB2* mutation but no family history of breast cancer. The patient’s father and sister were healthy carriers of the mutation, indicating parental heredity of a germline *PALB2* heterozygote mutation.

TABLE S1 Germline and somatic mutations in 18 patients with familial cancers other than FBCs and heterozygous germline *PALB2* mutations.

TABLE S2, S3 Analysis of loss of heterozygosity at the *PALB2* locus in 28 mono-allelic *PALB2* mutant familial cancers patients. Colon cancer (ID5), pulmonary cancer (ID10), breast cancer (ID20 and ID28) had LOH. All this patients were highlighted in red color in Supplementary Table S2.

TABLE S4 TMB and Prognostic imformation about *PALB2* mutant patients. TMB^∗^ (mut/MB), tumor mutation burden, was divided into three levels (U, Low and High), according to the protocol (Hu et al., 2018). TMB-U represented the variated allele (single nucleotide variants, small insertion and deletion) frequency <3%. TMB-high patients were identified with ≥11 mut/MB (upper quartile of all data). Others were identified as TMB-low patients. Progression^∗∗^ (Owens et al., 2019) identified patients who recieved standard treatment but progressed within 6 months after the genetic testing; otherwise, progression = 0.

TABLE S5 Increased cancer risk in carriers of the *PALB2* c.3114-1G > A mutation.

TABLE S6 Charger summary of the detected *PALB2* variants.
