# Supplementary figures and images for: Germline PALB2 Mutations in Cancers and Its Distinction From Somatic PALB2 Mutations in Breast Cancers
Source: Front Genet. 2020 Aug 27;11:829. doi: 10.3389/fgene.2020.00829 (PMC7482549; doi:10.3389/fgene.2020.00829)

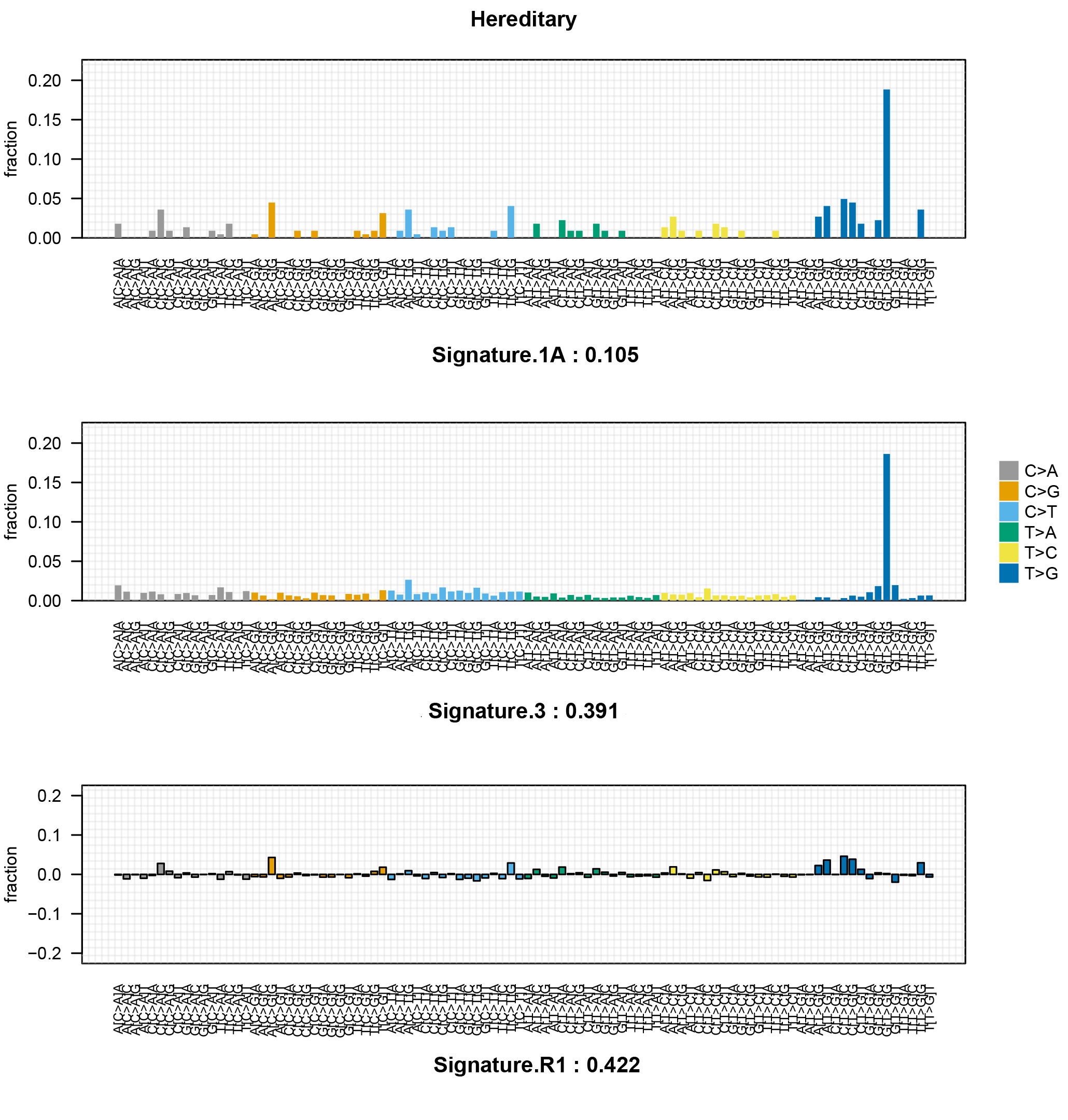

Supplement: FIGURE S1 — Frequency plots of mutational signature of hereditary PALB2 tumors. The proportions of signature 1A (top), signature 3 (medium), and signature R1 (bottom) were 10.5%, 39.1%, and 42.2%, respectively. [file Image_1.TIF]

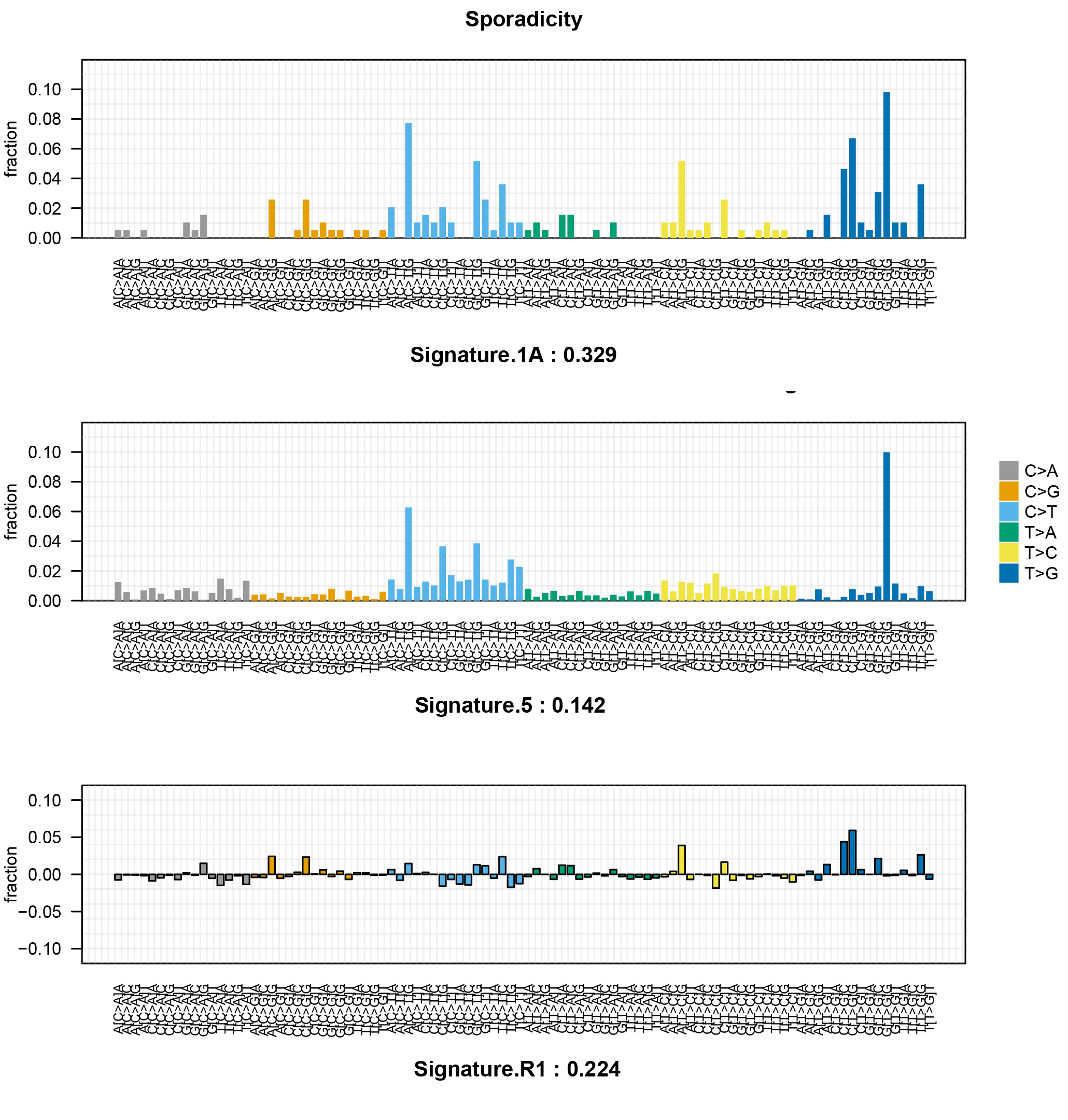

Supplement: FIGURE S2 — Frequency plots of mutational signature of sporadic breast tumors. The proportions of signature 1A (top), signature 5 (medium), and signature R1 (bottom) were 32.9%, 14.2%, and 22.4%, respectively. [file Image_2.TIF]

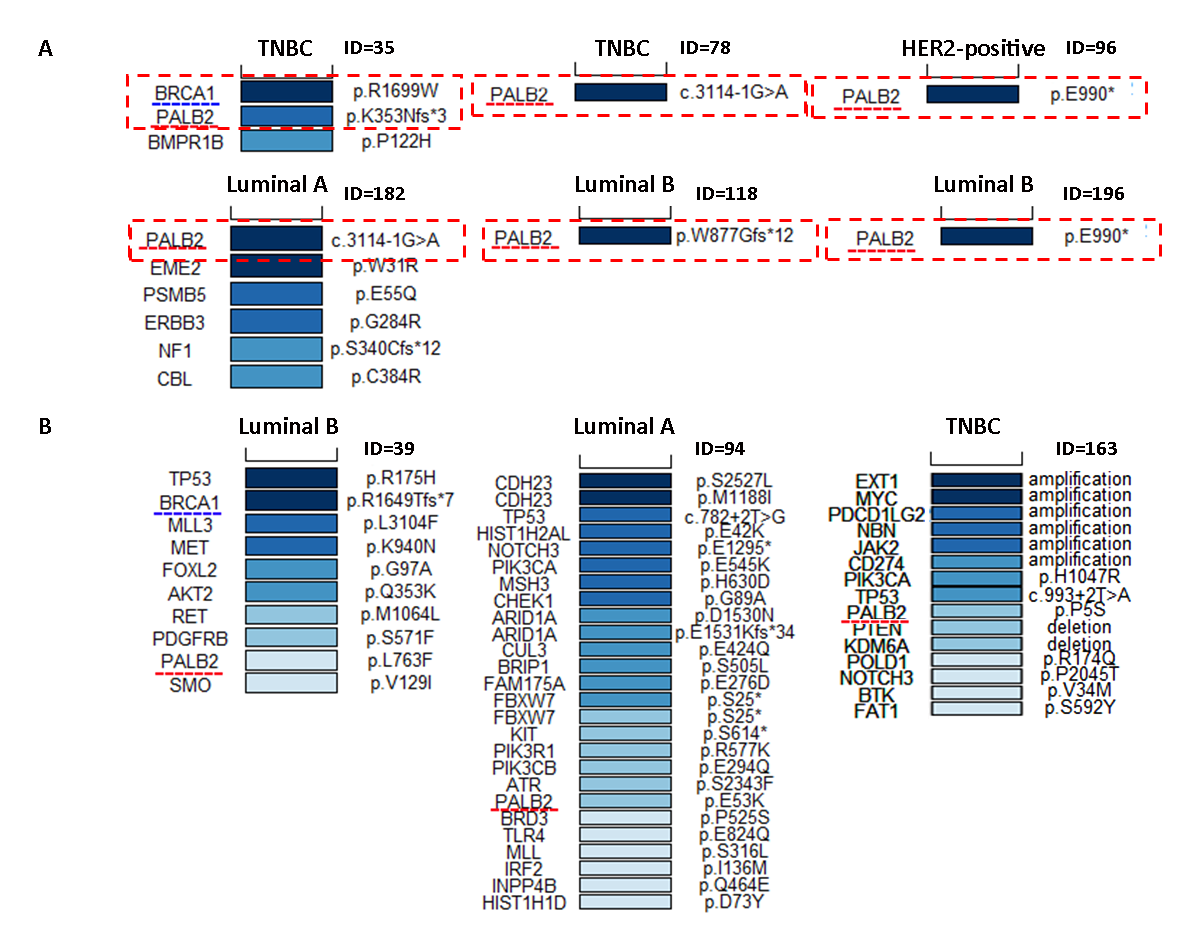

Supplement: FIGURE S3 — Germline (in the red dash rectangles) and somatic mutation profiles of advanced breast cancers with germline or somatic PALB2 mutation. BRCA1 (blue dash line) and PALB2 (red dash line) mutations were highlighted. Dark blue represents the most commonly mutated genes, and light blue represents the least commonly mutated genes. If genes were mutated at the same frequency, they are listed in alphabetic order. (A) Among 196 advanced breast cancers, two triple-negative breast cancer (TNBC) tumors (ID35, ID78), two Luminal B (ID118, ID196), one Luminal A (ID182) and one Her2-positive tumors (ID96) had mono-allelic germline PALB2 mutation. (B) One TNBC (ID163), one Luminal A (ID94) and one Luminal B (ID39) advanced breast cancers had somatic PALB2 mutations. [file Image_3.TIF]

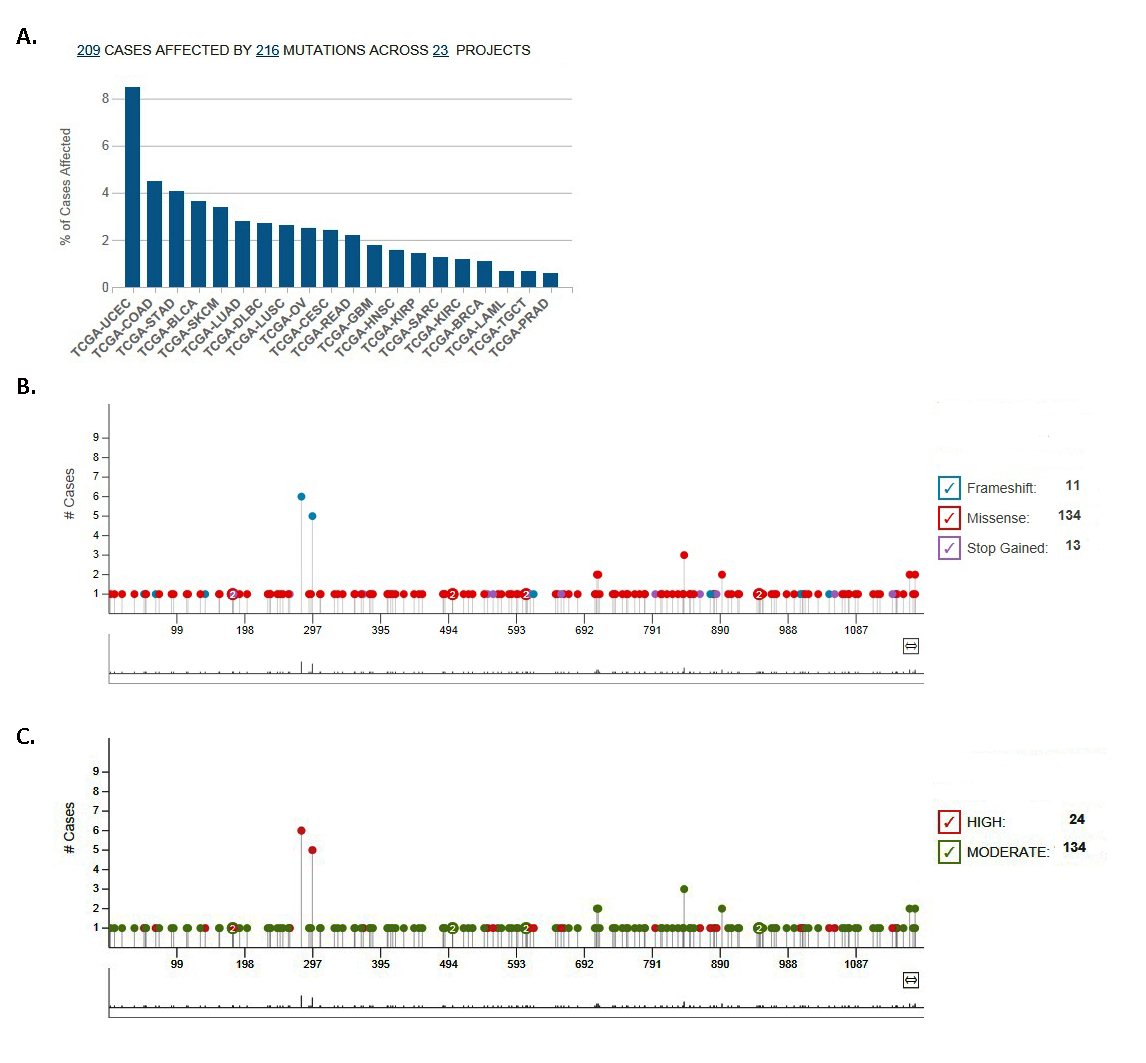

Supplement: FIGURE S4 — Somatic PALB2 mutations detected in the TCGA database. (A) Percentages of patients affected by a total of 216 PALB2 mutations across 23 projects in the TCGA database. A total of 209 patients were affected by PALB2 mutations. Somatic PALB2 mutations were detected in 8.09% of patients with uterine corpus endometrial carcinoma and 1.12% of patients with invasive breast carcinoma (TCGS-BRCA). (B) Numbers of patients affected by each of 158 somatic PALB2 variations in the TCGA database. A total of 209 patients were affected by PALB2 mutations. There were 134 missense mutations (red dots). The frameshift mutations (blue dots) p.N280Tfs∗8 and p.M296∗ were the most frequent mutations, affecting six and five patients, respectively). (C) The risk level associated with each mutation in B. The Frameshift and stop-gain mutations were high-risk PALB2 variations (red dots). Missense mutations were moderate-risk variations (blue dots). [file Image_4.TIF]

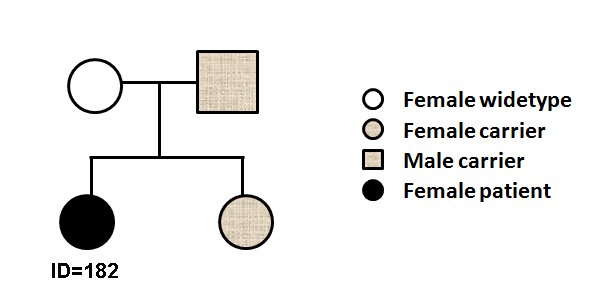

Supplement: FIGURE S5 — Pedigree for patient ID182. This patient had a heterozygous germline PALB2 mutation but no family history of breast cancer. The patient’s father and sister were healthy carriers of the mutation, indicating parental heredity of a germline PALB2 heterozygote mutation. [file Image_5.TIF]
