# Supplementary material for: Germline PALB2 Mutations in Cancers and Its Distinction From Somatic PALB2 Mutations in Breast Cancers
Source: Front Genet. 2020 Aug 27;11:829. doi: 10.3389/fgene.2020.00829 (PMC7482549; doi:10.3389/fgene.2020.00829)
Supplement: TABLE S1 — Germline and somatic mutations in 18 patients with familial cancers other than FBCs and heterozygous germline PALB2 mutations. [file Table_1.DOCX]

**Table S1**. Germline and somatic mutations in 18 patients with familial cancers other than FBCs and heterozygous germline *PALB2* mutations.

| **ID** | **Cancer type** | **germline mutation** | |  | **Somatic mutations** | |
| --- | --- | --- | --- | --- | --- | --- |
|  |  | **PALB2** | ***BRCA1/2*** |  | ***TP53*** | **Other** |
| 1 | Stomach carcinoma | c.3114-2A>G[splicing] **^Ϯ^** | . |  | c.536A>G[p.H179R] | *KRAS/IGFR1/PTCH2* |
| 2 | Pancreatic carcinoma | c.2108T>A[p.L703*] **^Ϯ Ϯ^** |  |  |  | *SMAD2/IDH1/PTCH1* |
| 3 | Cholangiocarcinoma | c.2594C>G[p.S865*] **^Ϯ Ϯ^** |  |  | c.818G>A[p.R273H] | *KRAS* |
| 4 | Colon Adenocarcinoma | c.2257C>T[p.R753*] **^Ϯ^** |  |  | c.811G>A[p.E271K] | *PIK3CA/KRAS/APC* |
| 5 | Colon Adenocarcinoma | c.2297_2298dupCA[p.V767Qfs*85] **^Ϯ Ϯ^** |  |  |  | *PIK3CA/KRAS/APC* |
| 6 | Rectal Adenocarcinoma | c.2693G>A[p.W898*] **^Ϯ Ϯ^** |  |  | c.534C>A[p.H178Q] | \| *PALB2[*c.1834_1843del(p.F612Lfs*13)]**^ϮϮ^** \| \| --- \|   */PIK3R2/PTEN* |
| 7 | Rectal Adenocarcinoma | c.2869A>T[p.K957*] **^Ϯ Ϯ^** |  |  |  | *APC/BRAF/MAP2K4* |
| 8 | Rectal Adenocarcinoma | c.2968G>T[p.E990*] **^Ϯ Ϯ^** |  |  | c.707A>G[p.Y236C] | *APC/XRCC2/TCF7L2* |
| 9 | Pulmonary Carcinoma | c.7G>T[p.E3*] **^Ϯ Ϯ^** |  |  |  | *AKT2/ BRCA2/NRAS/RB1* |
| 10 | Pulmonary Carcinoma | c.751C>T[p.Q251*] **^Ϯ Ϯ^** |  |  | c.815T>G[p.V272G] | *CDKN2A* |
| 11 | Pulmonary Carcinoma | c.1037_1041delAAGAA[p.K346Tfs*13] **^Ϯ Ϯ^** |  |  | c.155_164del[p.Q52Lfs*68] | *EGFR* |
| 12 | Pulmonary Carcinoma | c.2167_2168delAT[p.M723Vfs*21] **^Ϯ^** |  |  |  | *EGFR/BRCA2/AR* |
| 13 | Pulmonary Carcinoma | c.2167_2168delAT[p.M723Vfs*21] **^Ϯ^** |  |  | c.949C>T[p.Q317*] | *EGFR/ERBB3/PDGFRA* |
| 14 | Pulmonary Carcinoma | c.2257C>T[p.R753*] **^Ϯ^** |  |  | c.277_278delCT[p.L93Vfs*55 | *NOTCH1* |
| 15 | Pulmonary Carcinoma | c.2760dupA[p.Q921Tfs*7] **^Ϯ Ϯ^** |  |  | c.412G>C[p.A138P] | *EGFR* |
| 16 | Pulmonary Carcinoma | c.2968G>T[p.E990*] **^Ϯ Ϯ^** |  |  |  | *ESR1/CHEK1* |
| 17 | Prostate cancer | c.472delC[p.Q158Rfs*19] **^Ϯ Ϯ^** | *BRCA2*: 8474_8487del[p.A2825Vfs*15] |  |  | *CDH11/FOXA1* |
| 18 | Prostate cancer | c.2968G>T[p.E990*] **^Ϯ Ϯ^** |  |  |  | *AR/PDGFRA* |

Note: Key somatic mutations were highlighted in red color; patients with mono-allelic *PALB2* germline mutation and without any other accompanied mutations were highlighted in yellow color.

**^Ϯ^** indicated reported mutations while **^Ϯ Ϯ^** indicated the novel mutations.
