# Supplementary material for: Germline PALB2 Mutations in Cancers and Its Distinction From Somatic PALB2 Mutations in Breast Cancers
Source: Front Genet. 2020 Aug 27;11:829. doi: 10.3389/fgene.2020.00829 (PMC7482549; doi:10.3389/fgene.2020.00829)
Supplement: TABLE S2, S3 — Analysis of loss of heterozygosity at the PALB2 locus in 28 mono-allelic PALB2 mutant familial cancers patients. Colon cancer (ID5), pulmonary cancer (ID10), breast cancer (ID20 and ID28) had LOH. All this patients were highlighted in red color in Supplementary Table S2. [file Table_2.DOCX]

**Table S2-S3**. Analysis of loss of heterozygosity at the *PALB2* locus in 28 mono-allelic *PALB2* mutant familial cancers patients.

**Table S2**.

| **ID** | **Sampleid** | **Cancer type** | **gene.symbol** | **chr** | **start** | **end** | **loh** |
| --- | --- | --- | --- | --- | --- | --- | --- |
| 1 | 180013549BCD_180013549BPD | Stomach carcinoma | PALB2 | 16 | 23614766 | 23652514 | FALSE |
| 2 | 170012669BCD_170012669TD | Pancreatic carcinoma | PALB2 | 16 | 23614766 | 23652514 | FALSE |
| 3 | 170013993BCD_170013993BPD | Cholangiocarcinoma | PALB2 | 16 | 23614766 | 23652514 | FALSE |
| 4 | 180009859BCD_180009859BPD | Colon Adenocarcinoma | PALB2 | 16 | 23614766 | 23652514 | FALSE |
| 5 | 180023746BCD_180023746BPD | Colon Adenocarcinoma | PALB2 | 16 | 23614766 | 23652514 | TRUE |
| 6 | 180015245BCD_180015245BPD | Rectal Adenocarcinoma | PALB2 | 16 | 23614766 | 23652514 | FALSE |
| 7 | 180000569BCD_180010427BPD | Rectal Adenocarcinoma | PALB2 | 16 | 23614766 | 23652514 | FALSE |
| 8 | 170017301BCD_170017301BPD | Rectal Adenocarcinoma | PALB2 | 16 | 23614766 | 23652514 | FALSE |
| 9 | 180000576BCD_180000576BPD | Pulmonary Carcinoma | PALB2 | 16 | 23614766 | 23652514 | FALSE |
| 10 | 180015842BCD_180015842BPD | Pulmonary Carcinoma | PALB2 | 16 | 23614766 | 23652514 | TRUE |
| 11 | 180013248BCD_180013248BPD | Pulmonary Carcinoma | PALB2 | 16 | 23614766 | 23652514 | FALSE |
| 12 | 170010464BCD_170010464BPD | Pulmonary Carcinoma | PALB2 | 16 | 23614766 | 23652514 | FALSE |
| 13 | 170016024BCD_170016029FD | Pulmonary Carcinoma | PALB2 | 16 | 23614766 | 23652514 | FALSE |
| 14 | 178000014BCD_178000014BPD | Pulmonary Carcinoma | PALB2 | 16 | 23614766 | 23652514 | FALSE |
| 15 | 170005644BD_170005644FD | Pulmonary Carcinoma | PALB2 | 16 | 23614766 | 23652514 | FALSE |
| 16 | 180010503BCD_180010503FD | Pulmonary Carcinoma | PALB2 | 16 | 23614766 | 23652514 | FALSE |
| 17 | 180007527BCD_180007527FD | Prostate cancer | PALB2 | 16 | 23614766 | 23652514 | FALSE |
| 18 | 180019905BCD_180019904FD | Prostate cancer | PALB2 | 16 | 23614766 | 23652514 | FALSE |
| 19 | 170015227BCD_170015227BPD | Breast Cancer | PALB2 | 16 | 23614766 | 23652514 | FALSE |
| 20 | 180020903BCD_180020902TD | Breast Cancer | PALB2 | 16 | 23614766 | 23652514 | TRUE |
| 21 | 180004534BCD_180004533TD | Breast Cancer | PALB2 | 16 | 23614766 | 23652514 | FALSE |
| 22 | 170016617BCD_170016624PD | Breast Cancer | PALB2 | 16 | 23614766 | 23652514 | FALSE |
| 23 | 180011822BCD_180011822BPD | Breast Cancer | PALB2 | 16 | 23614766 | 23652514 | FALSE |
| 24 | 170010001BCD_170010001BPD | Breast Cancer | PALB2 | 16 | 23614766 | 23652514 | FALSE |
| 25 | 170014194BCD_170014194BPD | Breast Cancer | PALB2 | 16 | 23614766 | 23652514 | FALSE |
| 26 | 180006546BCD_180006546BPD | Breast Cancer | PALB2 | 16 | 23614766 | 23652514 | FALSE |
| 27 | 180002559BCD_180008306FD | Breast Cancer | PALB2 | 16 | 23614766 | 23652514 | FALSE |
| 28 | 170015383BCD_170015383BPD | Breast Cancer | PALB2 | 16 | 23614766 | 23652514 | TRUE |

**Table S3**.

| ID | Sample | **Cancer type** | Chr | Pos | Ref | Alt | NormalFreq | CaseFreq |
| --- | --- | --- | --- | --- | --- | --- | --- | --- |
| 1 | 180013549BCD_180013549BPD | Stomach carcinoma | 16 | 23646191 | T | C | 48% | 42% |
| 2 | 170012669BCD_170012669TD | Pancreatic carcinoma | 16 | 23640467 | G | A | 53% | 54% |
| 2 | 170012669BCD_170012669TD | Pancreatic carcinoma | 16 | 23646191 | T | C | 51% | 53% |
| 4 | 180009859BCD_180009859BPD | Colon Adenocarcinoma | 16 | 23637777 | A | C | 49% | 49% |
| 5 | 180023746BCD_180023746BPD | Colon Adenocarcinoma | 16 | 23637777 | A | C | 48% | 41% |
| 5 | 180023746BCD_180023746BPD | Colon Adenocarcinoma | 16 | 23640467 | G | A | 45% | 59% |
| 5 | 180023746BCD_180023746BPD | Colon Adenocarcinoma | 16 | 23646191 | T | C | 51% | 57% |
| 6 | 180015245BCD_180015245BPD | Rectal Adenocarcinoma | 16 | 23640467 | G | A | 46% | 51% |
| 7 | 180000569BCD_180010427BPD | Rectal Adenocarcinoma | 16 | 23640467 | G | A | 53% | 49% |
| 8 | 170017301BCD_170017301BPD | Rectal Adenocarcinoma | 16 | 23646191 | T | C | 51% | 48% |
| 9 | 180000576BCD_180000576BPD | Pulmonary Carcinoma | 16 | 23646191 | T | C | 51% | 49% |
| 10 | 180015842BCD_180015842BPD | Pulmonary Carcinoma | 16 | 23640467 | G | A | 55% | 73% |
| 10 | 180015842BCD_180015842BPD | Pulmonary Carcinoma | 16 | 23646191 | T | C | 49% | 77% |
| 11 | 180013248BCD_180013248BPD | Pulmonary Carcinoma | 16 | 23640467 | G | A | 48% | 49% |
| 12 | 170010464BCD_170010464BPD | Pulmonary Carcinoma | 16 | 23640467 | G | A | 51% | 49% |
| 12 | 170010464BCD_170010464BPD | Pulmonary Carcinoma | 16 | 23641367 | A | T | 49% | 49% |
| 12 | 170010464BCD_170010464BPD | Pulmonary Carcinoma | 16 | 23647149 | G | T | 46% | 50% |
| 13 | 170016024BCD_170016029FD | Pulmonary Carcinoma | 16 | 23637711 | G | C | 44% | 55% |
| 13 | 170016024BCD_170016029FD | Pulmonary Carcinoma | 16 | 23640467 | G | A | 52% | 56% |
| 13 | 170016024BCD_170016029FD | Pulmonary Carcinoma | 16 | 23646191 | T | C | 49% | 55% |
| 14 | 178000014BCD_178000014BPD | Pulmonary Carcinoma | 16 | 23640467 | G | A | 48% | 50% |
| 14 | 178000014BCD_178000014BPD | Pulmonary Carcinoma | 16 | 23641218 | G | A | 50% | 49% |
| 15 | 170005644BD_170005644FD | Pulmonary Carcinoma | 16 | 23646191 | T | C | 43% | 48% |
| 15 | 170005644BD_170005644FD | Pulmonary Carcinoma | 16 | 23646813 | C | G | 48% | 49% |
| 17 | 180007527BCD_180007527FD | Prostate cancer | 16 | 23634417 | T | A | 44% | 47% |
| 18 | 180019905BCD_180019904FD | Prostate cancer | 16 | 23640467 | G | A | 45% | 44% |
| 20 | 180020903BCD_180020902TD | Breast Cancer | 16 | 23640467 | G | A | 45% | 68% |
| 20 | 180020903BCD_180020902TD | Breast Cancer | 16 | 23646191 | T | C | 51% | 67% |
| 20 | 180020903BCD_180020902TD | Breast Cancer | 16 | 23647116 | G | A | 51% | 73% |
| 21 | 180004534BCD_180004533TD | Breast Cancer | 16 | 23646191 | T | C | 48% | 57% |
| 22 | 170016617BCD_170016624PD | Breast Cancer | 16 | 23640467 | G | A | 52% | 30% |
| 24 | 170010001BCD_170010001BPD | Breast Cancer | 16 | 23641218 | G | A | 46% | 52% |
| 26 | 180006546BCD_180006546BPD | Breast Cancer | 16 | 23640467 | G | A | 54% | 47% |
| 28 | 170015383BCD_170015383BPD | Breast Cancer | 16 | 23640467 | G | A | 52% | 85% |
