# Supplementary material for: Germline PALB2 Mutations in Cancers and Its Distinction From Somatic PALB2 Mutations in Breast Cancers
Source: Front Genet. 2020 Aug 27;11:829. doi: 10.3389/fgene.2020.00829 (PMC7482549; doi:10.3389/fgene.2020.00829)
Supplement: TABLE S4 — TMB and Prognostic imformation about PALB2 mutant patients. TMB∗ (mut/MB), tumor mutation burden, was divided into three levels (U, Low and High), according to the protocol (Hu et al., 2018). TMB-U represented the variated allele (single nucleotide variants, small insertion and deletion) frequency <3%. TMB-high patients were identified with ≥11 mut/MB (upper quartile of all data). Others were identified as TMB-low patients. Progression∗∗ (Owens et al., 2019) identified patients who recieved standard treatment but progressed within 6 months after the genetic testing; otherwise, progression = 0. [file Table_3.DOCX]

**Table S4**. TMB and Prognostic imformation about *PALB2* mutant patients.

| ID | Subtype | Stage | *PALB2* mutation | Aberrations | TMB* (mut/MB) | Progression** |
| --- | --- | --- | --- | --- | --- | --- |
| 35 | TNBC | 3 | Germline | p.K353Nfs*3 | U | 0 |
| 78 | TNBC | 4 | Germline | c.3114-1G>A | U | 1 |
| 96 | HR-/HER2+ | 3 | Germline | p.E990* | U | 0 |
| 118 | HR+/HER2- | 4 | Germline | p.W877Gfs*12 | 1 (Low) | 0 |
| 182 | HR+/HER2- | 4 | Germline | c.3114-1G>A | 5 (Low) | 0 |
| 195 | HR+/HER2- | 4 | Germline | p.E990* | U | 0 |
| 39 | HR+/HER2- | 4 | Somatic | p.L763F | 14.4 (High) | 1 |
| 94 | HR+/HER2- | 4 | Somatic | p.E53K | 62 (High) | 1 |
| 163 | TNBC | 4 | Somatic | p.P5S | 8 (Low) | 1 |

Note: TMB* (mut/MB), tumor mutation burden, was divided into three levels (U, Low and High), according to the protocol (21). TMB-U represented the variated allele (single nucleotide variants, small insertion and deletion) frequency < 3 %,. TMB-high patients were identified with ≥11 mut/MB (upper quartile of all data). Others were identified as TMB-low patients.

Progression** (1) identified patients who recieved standard treatment but progressed within 6 months after the genetic testing; otherwise, progression=0.
