# Supplementary material for: Germline PALB2 Mutations in Cancers and Its Distinction From Somatic PALB2 Mutations in Breast Cancers
Source: Front Genet. 2020 Aug 27;11:829. doi: 10.3389/fgene.2020.00829 (PMC7482549; doi:10.3389/fgene.2020.00829)
Supplement: TABLE S5 — Increased cancer risk in carriers of the PALB2 c.3114-1G > A mutation. [file Table_4.DOCX]

**Table S5. Increased cancer risk in carriers of the *PALB2* c.3114-1G>A mutation**

| **Type of cancer** | **Lifetime risk for carrier** | **Life-time risk for non-carrier** |
| --- | --- | --- |
| Breast cancer (Female) | 33–58% | 12.4% |
| Pancreatic cancer | Increased | 0.5% |
| Breast cancer (Male) | Increased | 0.1% |
